# P-1966. Depression and Anxiety Symptoms During COVID-19: Age-Related Advantages in Well-Being

**DOI:** 10.1093/ofid/ofae631.2125

**Published:** 2025-01-29

**Authors:** Nicole S E Goh, Sharon Tan, Alex Yap, David Lye, Kelvin Bryan Tan

**Affiliations:** National Center of Infectious Diseases, Singapore, Singapore, Singapore; National Centre for Infectious Diseases, Singapore, Not Applicable, Singapore; National Center of Infectious Diseases, Singapore, Singapore, Singapore; National Centre for Infectious Diseases, Singapore, Not Applicable, Singapore; National Centre for Infectious Diseases / Ministry of Health, Singapore, Not Applicable, Singapore

## Abstract

**Background:**

The long-term effects of the COVID-19 pandemic on individuals’ physical and mental health are of widespread societal concern. However, in the sphere of mental health, little research has been done on the pandemic’s differential impacts on different sociodemographic groups over time.

**Methods:**

This study evaluates anxiety and depression symptoms across different age groups in Singapore as pandemic restrictions were gradually lifted. Cross-sectional data was gathered in 36 waves through the Imperial College London-YouGov Covid-19 Behavioral Tracker. Results from 34429 adults in Singapore between 27 April 2020 and 27 September 2021 were analyzed. Multivariable logistic and linear regression were carried out to assess Patient Health Questionnaire-4 (PHQ-4) scores over pandemic phases and by age, adjusting for sex, employment status, availability of COVID-19 vaccine, and pre-existing mental health conditions.

**Results:**

Overall depression and anxiety symptoms as measured by the PHQ-4 did not vary significantly across the pandemic. However, younger age groups reported higher PHQ-4 scores as restrictions lifted (ages 18-29: *d=0.59, 95% CI [0.22, 0.97], p< 0.05*; ages 30-39: *d=0.43, 95% CI [0.07, 0.79], p< 0.05*), while older adults reported lower scores over time (ages 50-59: *d=-0.45, 95% CI [-0.80, -0.10, p< 0.05;* ages 60 and above: *d=-0.38, 95% CI [-0.74, -0.02], p< 0.05*). This was driven by increases in symptoms of anxiety for younger adults and reductions in symptoms of both depression and anxiety for older adults.

**Conclusion:**

Among Singaporean adults, age-related advantages are observed in psychological well-being. Population resilience to the pandemic may be heterogenous, with more intervention required to psychologically support younger adults.

**Disclosures:**

All Authors: No reported disclosures

